# Effect of foliar and soil application of plant growth promoting bacteria on growth, physiology, yield and seed quality of maize under Mediterranean conditions

**DOI:** 10.1038/s41598-020-78034-6

**Published:** 2020-12-03

**Authors:** Aspasia Efthimiadou, Nikolaos Katsenios, Sofia Chanioti, Marianna Giannoglou, Nikola Djordjevic, George Katsaros

**Affiliations:** 1 Institute of Soil and Water Resources, Department of Soil Science of Athens, Hellenic Agricultural Organization-DEMETER, Sofokli Venizelou 1, Lykovrissi, 14123 Attica, Greece; 2 Institute of Technology of Agricultural Products, Hellenic Agricultural Organization-DEMETER, Sofokli Venizelou 1, Lykovrissi, 14123 Attica, Greece; 3 Agrounik d.o.o., Milana Uzelca 11, 11080 Zemun, Serbia

**Keywords:** Photosynthesis, Plant breeding, Plant development

## Abstract

The use of plant growth promoting bacteria (PGPB) as biostimulants favors the increase of crop productivity and the improvement of yield quality. The main objective of the present study was to investigate the effect of the PGPB biostimulants (*Azotobacter chroococcum*, *Bacillus subtilis*, *Bacillus megatherium* and their mixes) and the application method (foliar and soil) on the growth, the physiology, the yield and the quality of maize. The obtained results showed that *A. chroococcum* treatment increased the chlorophyll content up to 6.1%, the photosynthetic rate up to 18.4% and the transpiration rate up to 34.3%. The highest maize yields were performed by the treatments *B. megatherium* (244.67 g) and the mix of *A. chroococcum* and *B. subtilis* (1:1) (243.67 g) when applied on the soil. The *Soil* application of the PGPB resulted in increased yield of maize from 5.5 to 13.4% compared to *control* treatment. Concerning quality characteristics, *B. subtilis* treatment increased total solids content in harvested maize seeds by 92%, as well as crude fiber content by 46% compared to control. The results confirmed that the use of PGPB could contribute as a new cultivation practice for sustainable growth, productivity and quality of grain crops.

## Introduction

Different species and strains of beneficial bacteria have been used during the last decades as biostimulants in various plant species with very promising results. Popular bacteria used as biostimulants include species such as *Arthrobacter* spp., *Acinetobacter* spp., *Enterobacter* spp., *Ochrobactrum* spp., *Pseudomonas* spp., *Rhodococcus* spp. and *Bacillus* spp.^1,2^. The urge for more sustainable cultivation practices has led the researchers worldwide to investigate the ability of plant growth promoting bacteria (PGPB) to enhance plant growth, yield and crop quality. The majority of plant growth promoting bacteria are naturally located in the soil environment and usually migrate to the rhizosphere and rhizoplane, however some of them move to aerial parts of the plants^3^. The scientific data regarding the positive interaction of certain bacteria strains and plants are increasing. Researches are now oriented to find the ideal combinations of Soil–Plant-PGPR system^4^ in order to maximize the positive effects of PGPR. An important advantage of biostimulants is that they are commercially sold in formulas, which are ready to use after a simple dilution, using the standard farming machinery^5^.

The mechanism used by PGPB to enhance plant growth and yield is not clearly defined, but many researches have reported that certain species and strains of PGPB have the ability to produce growth regulators^6,7^, are responsible for N_2_ fixation^8,9^, they create antagonistic environment for phytopathogens^10^ and they can induce the solubilization of mineral phosphates^11^. An hypothesis concerning the function of biostimulants is that they enhance plant productivity as they interact with plant signaling processes and reduce the negative plant responses to non-lethal stress that all crop plants are experiencing to varying degrees^12^. Studies have revealed that plant growth promoting rhizobacteria change hormones or release hormones in plants, produce volatile organic compounds which promote plant growth, improve nutrient availability and the uptake of plants and enhance abiotic stress tolerance in them^4^. Concerning the effectiveness of the PGPB, the use of indigenous strains of bacteria should be considered. As it was found in a pot experiment with spring wheat, indigenous strains are adapted in the environmental conditions and are more competitive and more effective than non-indigenous strains^13^.

A method of application of the PGPB is the foliar one, where the plant growth promoting bacteria are sprayed over the leaves of the cultivation. The foliar application of biostimulants creates an unknown interaction between the plant leaf surface and the microorganism, which needs to be further investigated^14^. Pati and Chandra^15^ claim that the humid tropical climate of Eastern India is ideal for the survival of such microorganisms on the leaf surface. Spraying with nitrogen fixing bacteria in winter wheat cultivation improved the growth of plants and increased yield by 70% compared to the control, a result similar to the use of chemical fertilizers^15^. In a 2-year experiment in mulberry, the foliar application of nitrogen fixing bacteria (*Azotobacter*, *Azospirillum* and *Beijerinckia*) improved yield quality and specifically *Azotobacter* increased the leaf production^16^. Foliar application of *Pseudomonas* and *Bacillus* strains on two apple varieties (Starkimson and Granny Smith) during the full blossom and 60 days after the full blossom, stimulated plant growth and increased yield in a 2-year experiment^17^. In a recent field study, strains of *Azotobacter chroococcum* and *Azospirillum lipoferum*, when combined together in a foliar application, increased plant height, branch number, seed yield and oil yield in canola^8^.

Another method of application is the inoculation of bacteria either on the seeds before sowing, or on the roots of seedling, or with direct application on soil. Inoculation of plant growth promoting bacteria (*Azotobacter chroococcum*, *Azospirillum brasilense* and the mix of them) was found that improves the physiological and biochemical characteristics of pennyroyal and avoids the adverse effects of lack of water^18^. Moreover, the use of plant growth promoting bacteria has been found that confers disease resistance to the plants^19^. A recent study on Barettiere (a melon variety), in a soilless system of cultivation, indicated that even in the absence of soil, PGPB improved fruit yield and some physiological parameters of the plant^20^. Root inoculation of *Bacillus* on strawberry organic cultivation increased yield, growth and chemical elements concentration of the plant^21^. Strains of *Azotobacter* phylogenetically related with *Azotobacter chroococcum*, were inoculated in maize roots, under different salinity conditions and the results showed that the treatment of PGPB increased chlorophyll content at zero salinity, while shoot length and shoot dry weight was higher under the first level of salinity^22^. It has been reported that the application of other types of biostimulants based on extracts from agricultural by-products or microalgae, free amino acids and synthetic preparations on crop cultivation increased the quality characteristics in terms of protein and crude fiber content of the plant products^23–26^. Still there is limited knowledge on the effect of the application of PGPB on the nutritional value and the quality of the harvested plant products. Since maize is one of the three most economically significant cereal crop, the assessment of the applicability of PGPB on the cultivation of that crop is of great interest for the agricultural community.

The aim of this study was to investigate the effect of three different plant growth promoting bacteria (*Azotobacter chroococcum*, *Bacillus subtilis*, *Bacillus megatherium*) and two mixes of them, as well as the method of application (foliar or soil) of these bacteria on the cultivation of maize in certain ecological zone and soil conditions. A wide range of measurements including growth, physiology and yield was performed. The quality characteristics of corn seeds, derived from different PGPB treatments, were also studied.

## Materials and methods

### Experimental site and design

A field experiment was established at Oropos (38°18′N, 23°45′E, Altitude 45 m), in the Prefecture of Attica, Greece, from April till August 2019. Monthly temperature and total precipitation are given in Figure S1.

A corn hybrid (*Zea mays*, GW 8002, Spyrou SA, Athens, Greece) was used for the establishment of the experimental cultivation. The sowing has been conducted at 21^st^ April 2019.

The experiment had a completely randomized design (CRD) with three replications and 2*6 treatments. The two main factors of the experimental design were: the method of the application of the PGPB (foliar or soil) and the PGPB used as biostimulants. The PGPB used were: *Azotobacter chroococcum* (Strain: B002, pH: 6.7, CFU/ml: 4*10^9^, Concentration of auxin: 35.6 ppm), *Bacillus subtilis* (Strain: B004, pH: 6.4, CFU/ml: 7*10^9^, Concentration of auxin: 30 ppm), *Bacillus megatherium* (Strain: A004, pH: 7.2, CFU/ml: 6.4*10^9^, Concentration of auxin: 24 ppm), Mix A: *A. chroococcum* + *B. subtilis* [1:1 (v/v)] and Mix B: *A. chroococcum* + *B. megatherium* [1:1 (v/v)]). The selection of the mixtures was based to our scope to investigate the combination of species of different genus (one Azotobacter plus one Bacillus species). Non-treated plots were used as control. Every replication was consisted of an area of 12m^2^. The space between rows was 75 cm. The application rate of PGPB was 7 lt/ha. The solution of the PGPB treatments that was either foliar-sprayed or added to the soil near the sowing rows, diluted with tap water (1:100) in order to have a concentration of bacteria 10^7^ CFU/ml. The application of the PGPB was conducted once, exactly at 40 DAS (Days After Sowing). When the maize was foliar sprayed, droplet size was regulated in order to avoid rolling off of the leaf surface. For the application of PGPB to the soil, the solution was applied to the planting rows, close to the maize plants.

The soil sample was collected from four representative cores of the experimental field in depth of 0–30 cm. The analyses of Ca, Mg, K, Na were conducted according to ISO, 1994 (11,260), of Zn, Mn, Cu and Fe according to ISO, 2001 (14,870), the available B was determined using azomethine-H as the color development reagent, total Nitrogen according to ISO, 1995 (11,261), organic carbon according to ISO, 1998 (14,235), available Phosphorus with ISO, 1994 (11,263), Cation Exchange Capacity was determined according to ISO 11,260 (1994), soil texture was determined using the method of Bouyoucos, the moisture content was determined in a furnace at 105° C for 24 h and the value of pH was measured with a pH-meter in the saturated paste extract. The soil analysis was performed a few days before sowing, and the results indicated that there were sufficient quantities of all basic micro and macro nutrients (Table 1).

**Table 1:** Physical and chemical properties of soil.

Parameters	Values
Sand (%)	36
Silt (%)	28
Clay (%)	36
Soil texture	Clay Loam
pH	7.5
Saturation percentage (%)	60
Electrical conductivity (mS/cm)	1.57
Total salts (%)	0.06
Organic matter (%)	5.5
Total nitrogen (mg/g)	2.8
CaCO_3_ (%)	14
Available K (cmoℓ + /kg)	1.1
Available Ca (cmoℓ + /kg)	27
Available Mg (cmoℓ + /kg)	7.3
Available P (mg/kg)	99
Available Fe (mg/kg)	22
Available Cu (mg/kg)	4.1
Available Zn (mg/kg)	8.2
Available Mn (mg/kg)	22
Available B (mg/kg)	0.9

### Cultivation of bacteria

The bacterial strains were isolated from an agricultural soil, that was cultivated with maize, by the streaking method. Twelve bacterial strains were isolated, among which were Bacillus subtilis, Bacillus megatherium and Azotobacter chroococcum. The bacteria were identified by sequencing 16 rDNA. Individual colonies were seeded in 100 ml of TSB and *Azotobacter* medium for 24 h, and optical density was between 0.3–0.35. After that, 2% of the inoculum was seeded in 3L of the medium. *Bacillus subtilis* and *Bacillus megatherium* were cultivated in Tryptic soy broth (TSB) and grow under aerobic condition at 32 °C with shaking at 200 rpm^27^ for 48 h. *Azotobacter chroococcum* was cultivated in *Azotobacter* medium and grew at 30 °C with shaking at 180 rpm for 72 h. On the end of fermentation, bacterial strains were tested for the optimal growth, pH and production of plant hormone auxin by colourimetric analysis^28^. After that, *A. Chroococcum* was mixed with *B. subtilis* and *B. megatherium* in the relative 1:1^29^.

### Measurements

The agronomic and physiology measurements (dry weight, chlorophyll content, photosynthetic rate, transpiration rate and stomatal conductance) were measured 52, 72 and 88 DAS. Dry weight (g per plant) of the shoots was a destructive measurement, three plants per plot were collected from the middle lines and they were measured after they were oven dried at 70 °C for three days in a precision balance. For the determination of the chlorophyll (μg/cm^2^) content, a portable chlorophyll meter (SPAD) was used. The physiology measurements including photosynthetic rate (μmol CO_2_ m^−2^ s^−1^), transpiration rate (mmol H_2_O m^−2^ s^−1^) and stomatal conductance (mol m^−2^ s^−1^) were performed on sunny days, during midday hours on fully expanded leaves. Measurements were made using an LCi Leaf Chamber Analysis System (ADC, Bioscientific, Hoddesdon, UK). For the chlorophyll content, photosynthetic rate, transpiration rate and stomatal conductance, three measurements per plot were conducted. The yield measurement has been conducted at 20th August 2019 (122 DAS). Ten plants per plot were harvested and then a measurement of moisture content was conducted with a portable humidity meter and the yield results were adjusted to 15% humidity.

In order to examine the effect of the different PGPB treatments and their method of application on the quality of the harvested corn kernels, their essential physicochemical characteristics including size, sphericity, texture, total solids, total protein and total crude fiber content were determined. The corn seeds were harvested and dried in the shade. The moisture content of corn seeds before and after the drying procedure was approximately 15% and 8% w/w, respectively. Size is defined as the geometric mean diameter. The tri-axial dimensions including length, width and thickness of the corn seed were measured by a digital raider micrometer screw gauge to calculate its geometric mean diameter and the sphericity using Eqs. 1, 2.

1 $$ {\text{Size }} = \, \left( {{\text{a}}\cdot{\text{b}}\cdot{\text{c}}} \right)^{{{1}/{3}}} $$

2 $$ {\text{Sphericity}} = \left[ {\left( {{\text{a}}\cdot{\text{b}}\cdot{\text{c}}} \right)^{{{1}/{3}}} } \right]/{\text{a}} $$

where, a = length (mm); b = width (mm) c = thickness (mm).

Texture analysis was carried out by using a HD-Plus texture analyzer (Stable Micro Systems Ltd., UK) and the Texture Expert Exceed Software for the data analysis. The determination of the textural characteristics of corn seeds was conducted by a puncture probe of 5 mm diameter. Probe speeds of 1 mm/s during the test, 2 mm/s for pre- test and 10 mm/s for post- test were used throughout the study. All the measurements were performed at 25 ± 1 °C and the hardness of the corn seeds was determined. In order to be determined the total solids, total protein and total crude content, the corn seeds were ground by using a grinding mill. Total solids of corn flours were determined according to AOAC Official Method 925.09^30^. The total crude fibers of corn flours were determined by applying the Weende Method (AOAC 984.04) using a manual crude fiber analyzer (FibreBag-Fibretherm, Gerhardt analytical systems, Germany)^30^. Approximately 1 g of corn flour was weighed into glass tubes and hydrolyzed with boiling sulfuric acid (H_2_SO_4_) 0.26 N, followed by boiling in ΚΟΗ 0.23 Ν in a hot extractor beaker. After washing with distilled water and acetone, the residue was dried at 100 °C overnight followed by ignition in a muffle furnace at 450 °C for 5 h. Total protein content of corn flours was performed by applying the Kjeldahl method according to AOAC Official Method 920.87, using a Kjeldahl rapid distillation unit (Protein Nitrogen Distiller DNP-1500-MP, RAYPA, Spain)^30^. Approximately 1 g of corn flour was digested with 25 mL concentrated sulfuric acid (H_2_SO_4_) containing a mixture of copper sulfate and potassium sulfate at a ratio of 1:10 as catalyst at 400 °C for 3 h. After cooling, H_2_O was added and the ammonia samples were distilled into boric acid and titrated with hydrochloride acid 0.5 M to a colometric endpoint.

### Statistical analysis

A two-way analysis of variance (ANOVA) was used to evaluate the main effects of application method (Factor 1), PGPB (Factor 2) and the interaction between them. The experimental data were analyzed using IBM SPSS software ver. 24 (IBM Corp., Armonk, N.Y., USA). The comparisons of means were calculated using Duncan test at the 5% level of significance (*p* ≤ 0.05).

## Results and discussion

The use of plant growth promoting bacteria (PGPB) was found to have a positive effect on chlorophyll content, dry weight, photosynthetic rate, transpiration rate, stomatal conductance, yield, total solids, protein and crude fiber of maize. Moreover, it was found that the soil application of the PGPB gave better results than the foliar application. An interaction of the two factors (PGPB*application) was found at the measurement of photosynthetic rate for 72 DAS, yield and the total solids (Table 2).

**Table 2:** Analysis of variance (F values).

	Chlorophyll content (SPAD values)			Dry weight (g per plant)		
	52 DAS	72 DAS	88 DAS	52 DAS	72 DAS	88 DAS
PGPB	18.60***	3.80*	9.52***	14.89***	17.86***	11.81***
Application	24.68***	10.72**	41.00***	19.36***	23.73***	37.57***
PGPB*Application	1.23^ ns^	1.35^ ns^	2.15^ ns^	0.52^ ns^	0.61^ ns^	0.72^ ns^

	Photosynthetic rate (μmol CO_2_m^−2^ s^−1^)			Transpiration rate (mmol H_2_O m^−2^ s^−1^)		
	52 DAS	72 DAS	88 DAS	52 DAS	72 DAS	88 DAS
PGPB	16.29***	22.60***	4.07*	2.70*	2.15^ ns^	2.50^ ns^
Application	77.60***	131.50***	28.63***	12.83*	3.61^ ns^	4.41*
PGPB*Application	1.47^ ns^	4.09*	0.87^ ns^	0.05^ ns^	0.08^ ns^	0.03^ ns^

	Stomatal conductance (mol m^−2^ s^−1^)			Yield (kg per plant)		
	52 DAS	72 DAS	88 DAS			
PGPB	5.05**	2.31^ ns^	11.16***	3.41*		
Application	15.13**	11.36*	18.62***	90.91***		
PGPB*Application	0.41^ ns^	0.23^ ns^	0.63^ ns^	5.32*		

	Total solids	Protein	Crude fiber	Texture	Sphericity	Size
	(%)	(%)	(%)	(N)		(mm)
PGPB	120.13***	4.36**	56.64***	2.19^ ns^	0.75^ ns^	0.27^ ns^
Application	25.93***	0.09^ ns^	30.44***	0.05^ ns^	0.82^ ns^	0.01^ ns^
PGPB*Application	55.48***	2.30^ ns^	2.51^ ns^	1.60^ ns^	0.72^ ns^	0.90^ ns^

*. **. *** = significance at 0.05. 0.01 and 0.001.ns = not significant.

### Plant growth and physiology

The use of PGPB had a statistically significant effect on chlorophyll content (SPAD values) at all measurements (Table 3). At 52 DAS, the treatment with *A. chroococcum* gave the highest value (49.86) followed by *Mix B* (48.59) and *control* (47.18). At 72 DAS, all the treatments of PGPB had lower values than *control* (62.22), although these values were statistically significant different only compared to *Mix A* (58.00) and *B. subtilis* (55.74). At 88 DAS, the treatments with *Mix A* (60.67) and *A. chroococcum* (60.19) gave the highest values compared to all other treatments. Concerning the method of application, *soil* application of PGPB gave the higher values (49.97, 61.16 and 59.97) compared to the *foliar* application (42.37, 57.83 and 57.42) for 52, 72 and 88 DAS, respectively (*p* < 0.05).

**Table 3:** Influence of PGPB and their method of application on chlorophyll content 52, 72 and 88 DAS.

Treatments	Chlorophyll content (SPAD values)		
	52 DAS	72 DAS	88 DAS
**PGPB**			
*A. chroococcum*	49.86 ± 4.02 a	61.10 ± 3.23 ab	60.19 ± 2.13 a
*B. subtilis*	40.24 ± 4.56 c	55.74 ± 3.32 c	58.53 ± 1.74 b
*B. megatherium*	44.67 ± 3.73 b	58.76 ± 3.91 abc	58.56 ± 2.09 b
Mix A	37.50 ± 2.07 c	58.00 ± 3.04 bc	60.67 ± 2.34 a
Mix B	48.59 ± 4.64 a	61.15 ± 4.86 ab	57.02 ± 1.91 b
Control	47.18 ± 2.80 ab	62.22 ± 2.79 a	57.16 ± 0.75 b
**Application**			
Foliar	42.37 ± 4.77 b	57.83 ± 3.91 b	57.42 ± 1.46 b
Soil	46.97 ± 5.75 a	61.16 ± 3.46 a	59.97 ± 2.18 a

DAS: Days After Sowing. Mix A: *A. chroococcum* + *B. subtilis* (1:1), Mix B: A. *chroococcum* + *B. megatherium* (1:1). Means followed by the same letter for treatments are not significantly different according to Duncan test at *p* < 0.05. Values presented are mean values of three replicates ± standard deviation.

Specifically at the final measurement of the chlorophyll content, the treatment of *A. chroococcum* and *Mix A* that contains *A. chroococcum* and *B. megatherium*, gave higher values (5.3 and 6.1%, respectively) compared to *control*. The same species, *Azotobacter chroococcum*, in maize cultivation under saline stress, increased chlorophyll content 4–6 times compared to control^22^. Moreover, bacterial strains including *Azotobacter chroococcum* and *Bacillus megatherium*, increased chlorophyll content of *Hibiscus sabdariffa*^31^.

Dry weight measurements showed statistically significant differences in both use of PGPB and method of application (*p* < 0.05) (Table 4). In the first two measurements, the highest values in dry weight were measured in *A. chroococcum* (235.83 g and 581.09 g for 52 and 72 DAS, respectively) and *B. subtilis* (215.76 g and 566.76 g for 52 and 72 DAS, respectively) treatments with statistically significant differences compared to all other treatments. At the third measurement, the highest values were found at the treatments with *A. chroococcum* (609.33 g), *B megatherium* (598.95 g) and *Mix B* (597.38 g). All PGPB treatments gave statistically significant higher values compared to *control* (512.50 g), except from the treatment *Mix A*. Concerning the method of application, the *soil* application of PGPB gave the higher values (191.94, 539,61 and 597.11 g) compared to the *foliar* application (150.09, 497.62 and 540.19 g) for 52, 72 and 88 DAS, respectively.

**Table 4:** Influence of PGPB and their method of application on dry weight 52, 72 and 88 DAS.

Treatments	Dry weight (g per plant)		
	52 DAS	72 DAS	88 DAS
**PGPB**			
*A. chroococcum*	235.83 ± 34.94 a	581.09 ± 35.61 a	609.33 ± 59.15 a
*B. subtilis*	215. 76 ± 51.13 a	566.76 ± 49.84 a	555.26 ± 38.36 b
*B. megatherium*	134.33 ± 28.60 b	481.77 ± 31.10 b	598.95 ± 43.09 a
Mix A	123.93 ± 31.74 b	474.67 ± 28.77 b	538.47 ± 39.72 bc
Mix B	156.57 ± 31.08 b	504.23 ± 24.81 b	597.38 ± 37.81 a
Control	159.68 ± 29.25 b	503.18 ± 25.12 b	512.50 ± 16.83 c
**Application**			
Foliar	150.09 ± 47.32 b	497,61 ± 46.05 b	540.19 ± 37.02 b
Soil	191.94 ± 50.99 a	539.61 ± 49.57 a	597.11 ± 50.85 a

DAS: Days After Sowing. Mix A: *A. chroococcum* + *B. subtilis* (1:1), Mix B: *A. chroococcum* + *B. megatherium* (1:1). Means followed by the same letter for treatments are not significantly different according to Duncan test at *p* < 0.05. Values presented are mean values of three replicates ± standard deviation.

The use of PGPB increased dry weight for 88 DAS by 5.1–18.9% compared to the *control* treatment. Similar results, in maize and other cultivations have been found from many researchers. In a recent study, *Bacillus subtilis* treatments increased plant dry weight compared to the non fertilized control treatment on maize and sorghum cultivations^32^. Shoot dry weight of maize was 81–122% higher at the treatments that were used strains of *A. chroococcum* under high saline conditions in soil^22^. In a pot experiment in Columbia, the use of two strains of *Azotobacter chroococcum* in cotton, improved shoot length, root length, shoot dry weight, root dry weight, boll dry weight and N content^9^. The use of a mix of PGPB in two lettuce varieties, increased fresh weights in both spring and summer crops up to 18.9% and 22.7%, respectively^33^.

At the first measurement (52 DAS) of the photosynthetic rate, all PGPB treatments gave values statistically significant higher than *control* treatment (Table 5). Moreover, the treatment with *Mix B* (28.56 μmol CO_2_ m^−2^ s^−1^) had statistically significant differences compared to all other PGPB treatments (*p* < 0.05). At the second measurement, *control* treatment (31.80 μmol CO_2_ m^−2^ s^−1^) gave the lowest values with statistically significant differences compared to the PGPB treatments. Treatment of *Mix B* (37.36 μmol CO_2_ m^−2^ s^−1^) gave statistically significant differences compared to the other PGPB treatments, except for *B. subtilis* treatment (37.10 μmol CO_2_ m^−2^ s^−1^). At the third measurement, the highest values were measured in *B. megatherium* (34.96 μmol CO_2_ m^−2^ s^−1^) and *A. chroococcum* treatments (32.97 μmol CO_2_ m^−2^ s^−1^), with no statistically significant differences among them, whereas the treatments with *B. megatherium* had statistically significant differences compared to all other treatments. Concerning the method of application, the *soil* application of PGPB gave the higher values (27.67, 37.54 and 33.97 μmol CO_2_ m^−2^ s^−1^) compared to the *foliar* application (23.71, 33.61 and 30.41 μmol CO_2_ m^−2^ s^−1^) for 52, 72 and 88 DAS, respectively (*p* < 0.05).

**Table 5:** Influence of PGPB and their method of application on photosynthetic rate 52, 72 and 88 DAS.

Treatments	Photosynthetic rate (μmol CO_2_m^−2^ s^−1^)		
	52 DAS	72 DAS	88 DAS
**PGPB**			
*A. chroococcum*	25.75 ± 2.60 b	35.36 ± 2.59 c	32.97 ± 2.75 ab
*B. subtilis*	26.16 ± 2.68 b	37.10 ± 2.79 ab	32.42 ± 2.44 b
*B. megatherium*	25.51 ± 2.97 b	35.90 ± 2.65 bc	34.96 ± 2.60 a
Mix A	26.41 ± 2.87 b	35.92 ± 2.63 bc	31.46 ± 2.98 b
Mix B	28.56 ± 2.58 a	37.36 ± 2.68 a	30.68 ± 2.97 b
Control	21.74 ± 1.35 c	31.80 ± 1.30 d	30.65 ± 2.74 b
**Application**			
Foliar	23.71 ± 1.85 b	33.61 ± 1.42 b	30.41 ± 2.14 b
Soil	27.67 ± 2.93 a	37.54 ± 2.79 a	33.97 ± 2.62 a

DAS: Days After Sowing. Mix A: *A. chroococcum* + *B. subtilis* (1:1), Mix B: *A. chroococcum* + *B. megatherium* (1:1). Means followed by the same letter for treatments are not significantly different according to Duncan test at *p* < 0.05. Values presented are mean values of three replicates ± standard deviation.

Concerning the transpiration rate measurements (Table 6), the factor of PGPB treatments gave statistically significant differences only at 52 DAS, where *A. chroococcum* (3.41 mmol H_2_O m^−2^ s^−1^) gave the highest values with statistically significant differences compared to the treatment with *Mix A* (2.50 mmol H_2_O m^−2^ s^−1^) and *control* (2.54 mmol H_2_O m^−2^ s^−1^), while the differences with the rest of the treatments were not statistically significant.

**Table 6:** Influence of PGPB and their method of application on transpiration rate 52, 72 and 88 DAS.

Treatments	Transpiration rate (mmol H_2_O m^−2^ s^−1^)		
	52 DAS	72 DAS	88 DAS
**PGPB**			
*A. chroococcum*	3.41 ± 0.65 a	5.02 ± 0.80 a	3.69 ± 0.37 a
*B. subtilis*	3.01 ± 0.85 ab	4.76 ± 0.81 a	4.05 ± 0.44 a
*B. megatherium*	3.18 ± 0.62 ab	4.01 ± 0.97 a	4.16 ± 0.88 a
Mix A	2.50 ± 0.51 b	4.80 ± 0.38 a	4.29 ± 0.84 a
Mix B	3.13 ± 0.39 ab	4.39 ± 1.02 a	3.81 ± 0.53 a
Control	2.54 ± 0.60 b	5.40 ± 0.49 a	3.20 ± 0.16 a
**Application**			
Foliar	2.63 ± 0.56 b	4.47 ± 0.82 a	3.65 ± 0.58 b
Soil	3.30 ± 0.61 a	4.99 ± 0.83 a	4.08 ± 0.68 a

DAS: Days After Sowing. Mix A: *A. chroococcum* + *B. subtilis* (1:1), Mix B: *A. chroococcum* + *B. megatherium* (1:1). Means followed by the same letter for treatments are not significantly different according to Duncan test at *p* < 0.05. Values presented are mean values of three replicates ± standard deviation.

Concerning the method of application, the *soil* application of PGPB gave higher values with statistically significant differences compared to the *foliar* application at 52 and 88 DAS (*p* < 0.05).

As far as the stomatal conductance is concerned (Table 7), the use of PGPB gave statistically significant differences at 52 and 88 DAS, while the method of application gave statistically significant differences at all three measurements (*p* < 0.05). At the first measurement, *Mix A* (0.173 mol m^−2^ s^−1^) and *A. chroococcum* (0.168 mol m^−2^ s^−1^) treatments gave the highest values with statistically significant differences compared to *control*. At the third measurement, the treatments of *B. subtilis* (0.337 mol m^−2^ s^−1^), *Mix A* (0.332 mol m^−2^ s^−1^), *Mix B* (0.327 mol m^−2^ s^−1^) and *A. chroococcum* (0.315 mol m^−2^ s^−1^) gave the highest values with statistically significant differences compared to *control* (0.265 mol m^−2^ s^−1^) and *B. megatherium* (0.273 mol m^−2^ s^−1^) treatments. Concerning the method of application, the *soil* application of PGPB gave higher values (0.159, 0.305 and 0.324 mol m^−2^ s^−1^) compared to the *foliar* application (0.126, 0.272 and 0.292 mol m^−2^ s^−1^) for 52, 72 and 88 DAS, respectively.

**Table 7:** Influence of PGPB and their method of application on stomatal conductance 52, 72 and 88 DAS.

Treatments	Stomatal conductance (mol m^−2^ s^−1^)		
	52 DAS	72 DAS	88 DAS
**PGPB**			
*A. chroococcum*	0.168 ± 0.039 ab	0.302 ± 0.029 a	0.315 ± 0.040 a
*B. subtilis*	0.135 ± 0.039 c	0.295 ± 0.042 a	0.337 ± 0.031 a
*B. megatherium*	0.113 ± 0.023 c	0.305 ± 0.046 a	0.273 ± 0.024 b
Mix A	0.173 ± 0.037 a	0.297 ± 0.020 a	0.332 ± 0.030 a
Mix B	0.140 ± 0.021 bc	0.273 ± 0.024 a	0.327 ± 0.026 a
Control	0.127 ± 0.008 c	0.258 ± 0.026 a	0.265 ± 0.005 b
**Application**			
Foliar	0.126 ± 0.030 b	0.272 ± 0.028 b	0.292 ± 0.029 b
Soil	0.159 ± 0.032 a	0.305 ± 0.033 a	0.324 ± 0.041 a

DAS: Days After Sowing. Mix A: *A. chroococcum* + *B. subtilis* (1:1), Mix B: *A. chroococcum* + *B. megatherium* (1:1). Means followed by the same letter for treatments are not significantly different according to Duncan test at *p* < 0.05. Values presented are mean values of three replicates ± standard deviation.

Photosynthetic rate and stomatal conductance was higher in alfalfa plants inoculated with PGPB compared to control under normal and salinity conditions^34^. The inoculation of *Herbaspirillum seropedicae* in a field experiment of maize resulted in increased values of photosynthesis rate, stomatal conductance and transpiration rate of plants compared to the control^35^.

### Yield

**Figure 1:**
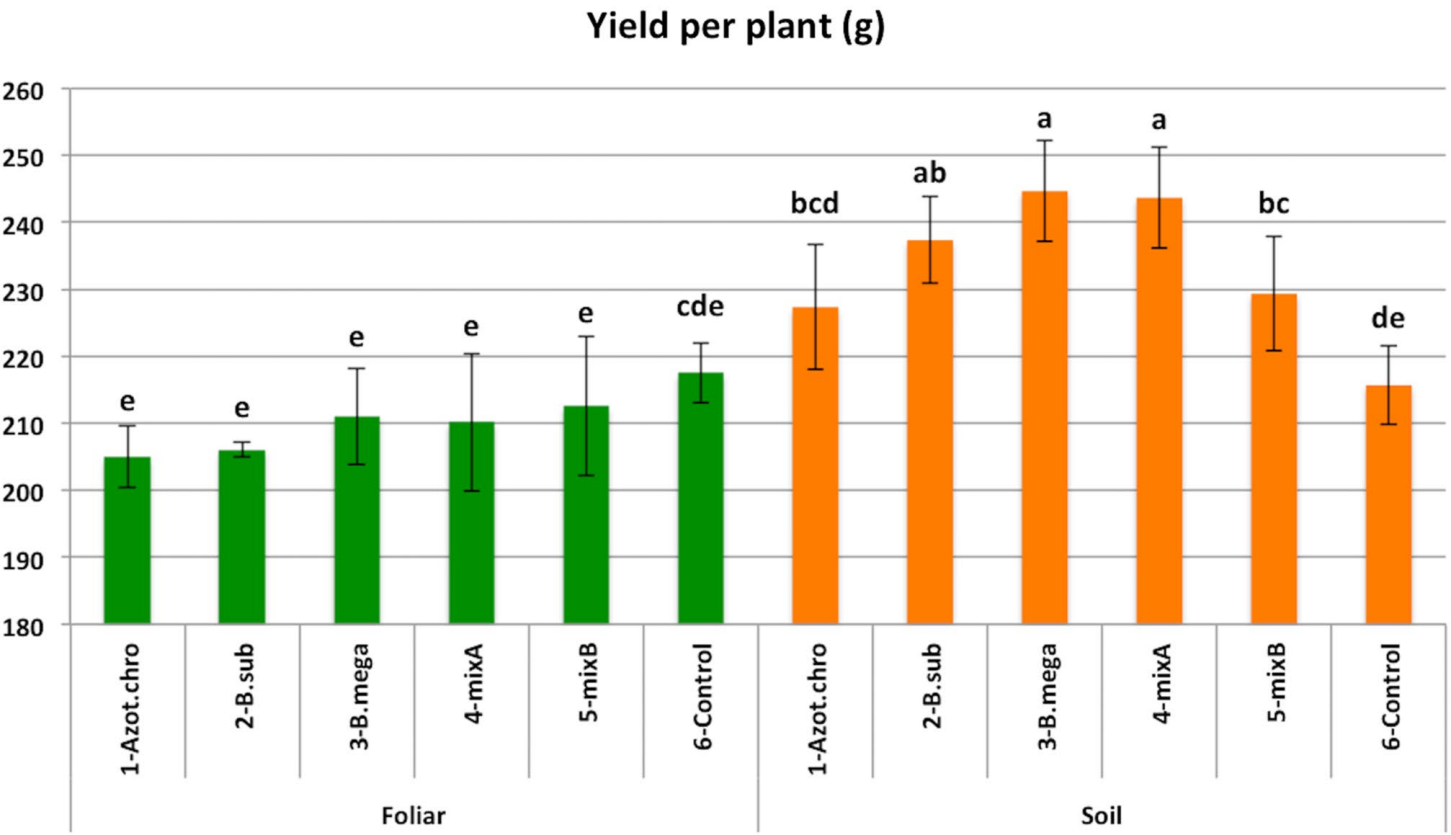
Interaction of five treatments of PGPB and two methods of application on the yield of maize. Means followed by the same letter for treatments are not significantly different according to Duncan test. Error bars are presenting standard deviation.

Yield measurements gave statistically significant differences. An interaction of the use of PGPB and the method of application was found (Fig. 1).

The highest values in yield (g per plant) were measured at *Soil-B. megatherium* (244.67 g) and *Soil-Mix A* (243.67 g) treatments with statistically significant differences compared to all treatments, except for *Soil-B. subtilis* (237.33 g) treatment (*p* < 0.05). *Soil-Mix B* (229.33 g) gave values with no statistically significant differences compared to *A. chroococcum* (227.33 g), whereas they were statistically significant different compared to the *Soil-control* (215.67 g). The values of the treatments of *Soil-control* and *Foliar-control* (217.46) were not statistically significant different. All the treatments of *Foliar* application of the PGPB gave values that were lower than the *Foliar-control* treatment, although differences were not statistically significant.

The *Soil* application of the PGPB resulted in increased yield of maize from 5.5 to 13.4% compared to *control* treatment. The *foliar* application of PGPB, under certain Mediterranean conditions, had no positive effect in the yield. The PGPBs that were used in this study (individual and in mixes) were found to improve crop yield in many cultivations. In spring wheat cultivation, the use of *Azotobacter chroococcum* strains, increased grain yield by 80–97% compared to the *control* in the pot experiment under greenhouse conditions and by 18–84% in the field experiment^13^. The root inoculation of *Bacillus* and the floral and foliar spraying of *Pseudomonas* and *Bacillus* on strawberry plants increased yield by 10.5–33.2%^21^. The use of combinations of bacteria was investigated in several researches and the results showed positive effects on yield^16^. It has been proved that the combination of different PGPB strains in same cases could be more effective than the single use of them, but this fact doesn't mean that always the combinations are more effective, as there are results which indicate that some combinations are less effective and that means that more research is needed in order to investigate the interactions between them^36^. A mix of *Bacillus circulance* and *Bacillus megatherium* inoculated in cotton enhanced morphological attributes and increased yield by 33% and 15% with 50% and 100% of recommended dose fertilization respectively, compared to the *control*^37^.

### Quality characteristics of the harvested corn seeds

The interaction of PGPB and their method of application on total solids of the harvested corn seeds is presented in Table 8. *Foliar-B. subtilis* (2.86%) and *Soil-A. chroococcum* (2.78%) treatments affected significantly the total solids of the corn seeds compared to all other treatments (*p* < 0.05). Table 9 shows the effect of PGPB and their method of application on total protein and crude fiber content, texture, sphericity and size of corn seeds.

**Table 8:** Interaction of PGPB and their method of application on total solids of the corn seeds.

Treatment	Total solids (%)
Foliar-*A. chroococcum*	2.25 ± 0.05 b
Foliar-*B. subtilis*	2.86 ± 0.08 a
Foliar-*B. megatherium*	1.90 ± 0.07 d
Foliar-Mix A	1.60 ± 0.01 e
Foliar-Mix B	1.27 ± 0.13 f.
Foliar-Control	1.49 ± 0.10 e
Soil-*A. chroococcum*	2.78 ± 0.06 a
Soil-*B. subtilis*	2.08 ± 0.12 c
Soil-*B. megatherium*	1.94 ± 0.06 cd
Soil-Mix A	1.53 ± 0.14 e
Soil-Mix B	2.09 ± 0.10 bc
Soil-Control	1.90 ± 0.11 d

DAS: Days After Sowing. Mix A: *A. chroococcum* + *B. subtilis* (1:1), Mix B: *A. chroococcum* + *B. megatherium* (1:1). Means followed by the same letter for treatments are not significantly different according to Duncan test at *p* < 0.05. Values presented are mean values of three replicates ± standard deviation.

**Table 9:** Influence of PGPB and their method of application on protein, crude fiber, texture, sphericity and size of corn seeds.

Treatments	Protein (%)	Crude fiber (%)	Texture (N)	Sphericity	Size (mm)
**PGPB**					
*A. chroococcum*	9.52 ± 1.01 a	4.45 ± 0.31 a	21.95 ± 2.44 a	0.637 ± 0.021 a	6.998 ± 0.113 a
*B. subtilis*	9.85 ± 1.15 a	4.73 ± 0.63 a	18.61 ± 2.60 a	0.659 ± 0.052 a	6.693 ± 0.233 a
*B. megatherium*	8.08 ± 0.72 b	3.21 ± 0.19 b	21.08 ± 2.73 a	0.674 ± 0.012 a	6.969 ± 0.119 a
Mix A	7.97 ± 1.18 b	2.79 ± 0.38 b	21.37 ± 3.00 a	0.682 ± 0.024 a	7.057 ± 0.291 a
Mix B	9.24 ± 1.45 ab	3.17 ± 0.34 b	20.72 ± 2.55 a	0.701 ± 0.041 a	7.075 ± 0.240 a
Control	9.99 ± 1.05 a	3.24 ± 0.31 b	23.24 ± 2.28 a	0.693 ± 0.024 a	6.816 ± 0.259 a
**Application**					
Foliar	9.16 ± 1.24 a	3.84 ± 0.82 a	21.06 ± 3.15 a	0.674 ± 0.012 a	7.007 ± 0.272 a
Soil	9.05 ± 1.44 a	3.36 ± 0.75 b	21.25 ± 2.51 a	0.678 ± 0.049 a	7.112 ± 0.177 a

DAS: Days After Sowing. Mix A: *A. chroococcum* + *B. subtilis* (1:1), Mix B: *A. chroococcum* + *B. megatherium* (1:1). Means followed by the same letter for treatments are not significantly different according to Duncan test at *p* < 0.05. Values presented are mean values of three replicates ± standard deviation.

The total protein content of corn seeds obtained *by control* (9.99%), *B. subtilis* (9.85%) and *A. chroococcum* (9.52%) treatments was significant higher than the one obtained by the other treatments, except for *Mix B* (9.24%) (*p* < 0.05). Corn seeds are one of the most valuable protein sources among the most important cereal crops and thus they are used in food products and livestock feeding. Similar observations were reported by Tejada et al.^38^, who found that by foliar-spraying maize crops with PGPB obtained from sewage sludges, the protein content of the harvested maize seeds was increased almost 30% in both tested growing seasons. Moreover, the protein content of maize seeds was increased by 19% by applying PGPB derived from olive oil by-product^39^. The foliar-spraying of soybean with high concentration of synthetic PGPB slightly increased the protein content in soybean seeds^23^.

By applying *B. subtilis* and *A. chroococcum* treatments, the harvested corn seeds contained the highest total crude fiber content (4.73% and 4.45%, respectively) of all the seeds of other treatments (*p* < 0.05). In particular, the crude fiber content of corn seeds was approximately 27% and 32% higher in *B. subtilis* and *A. chroococcum* treatments, respectively, compared to the *control*. The major fractions of crude fiber in grains include hemicellulose, cellulose and lignin; in particular, corn fiber consists of approximately 70% hemicellulose, 23% cellulose and 0.1% lignin (on dry basis)^24^. A high content of dietary fibers containing hemicelluloses is considered beneficial for the physiological processes on human organism, since they are able to absorb water and to promote the proliferation of gut microbiota in lumen^23^. The crude fiber content of soybean seeds was influenced by the application of biostimulants obtained from seaweed or amino acids in soybean cultivation^25^. As reported by Szparaga et al.^23^, the dietary fiber content of soybean seeds was increased by the application of the synthetic biostimulant Tytanit in soybean cultivation. When the biostimulants were applied foliar, the harvested corn seeds showed significant increased crude fiber content relative to the one obtained by soil application (*p* < 0.05) (Table 9). The foliar-spraying of biostimulants in plants affects the crude fiber content because of the increase of the gibberellins concentration altering the fiber formation mechanism^26^.

There were no significant differences in the texture, the size and the sphericity between corn grains from plants treated by PGPB and from untreated plants (Table 9). The fact that the application of PGPB improved significant the quality properties including total protein and crude fiber content of corn seeds without influencing their physical properties (texture, size) could be an advantage for the use of the current individual processes including harvesting, handling as well as preparation into food products^40^.

In general, soil application of PGPB provided better results in terms of quantity and quality of the kernel yield of maize under Mediterranean conditions. This is a very important finding, due to the fact that most of the researchers are mainly focused on testing many PGPB with one way of application method. Our results indicate that the method of application of the PGPB plays a crucial role on the effectiveness of the treatment. A possible reasoning for these results could be the better adoption and the longevity of the bacteria in the soil, instead of the leaves of the maize.

## Conclusions

The type of plant growth promoting bacteria (PGPB) used as biostimulants (*Azotobacter chroococcum*, *Bacillus subtilis*, *Bacillus megatherium* and their mixes) and the method of their application (foliar and soil) significantly affected the growth, physiology and crop yield of maize as well as the quality characteristics of the harvested corn seeds. It was demonstrated that the chlorophyll content was positively affected by *A. chroococcum* treatment and the dry weight was significantly influenced by all the PGPB treatments. In addition, a positive effect was detected by the application of these PGPB, and in particular *A. chroococcum,* on the photosynthetic rate, the transpiration rate and the stomatal conductance of maize plants. Also, the soil application increased significantly the chlorophyll content, the dry weight and the physiology measurements of maize compared to those obtained by foliar sprays. Tested PGPB, when applied in soil, enhanced the yield of maize; *B. megatherium* and the mix of *A. chroococcum* and *B. subtilis* (1:1) treatments resulted in the greatest maize productivity. Concerning quality characteristics, *B. subtilis* treatment increased total solids content in harvested maize seeds by 92%, as well as crude fiber content by 46% compared to control. The obtained results suggested that tested PGPB could be used as biostimulants in order to improve maize yield and quality, however further studies aiming to determine the mechanisms of action of such PGPB treatments on various cereal crops should be performed. In order to understand the activity of biostimulants on crop productivity and to provide practical recommendations for supporting the agricultural field, different parameters in terms of the biostimulants such as the type, the quantity, the timing and the number of application, as well as the type of crop should be examined and optimized.

## Supplementary information

Supplementary Information.
