# Supplementary material for: Effect of foliar and soil application of plant growth promoting bacteria on growth, physiology, yield and seed quality of maize under Mediterranean conditions
Source: Sci Rep. 2020 Dec 3;10:21060. doi: 10.1038/s41598-020-78034-6 (PMC7713431; doi:10.1038/s41598-020-78034-6)
Supplement: Supplementary file 1 — Supplementary Information. [file 41598_2020_78034_MOESM1_ESM.docx]

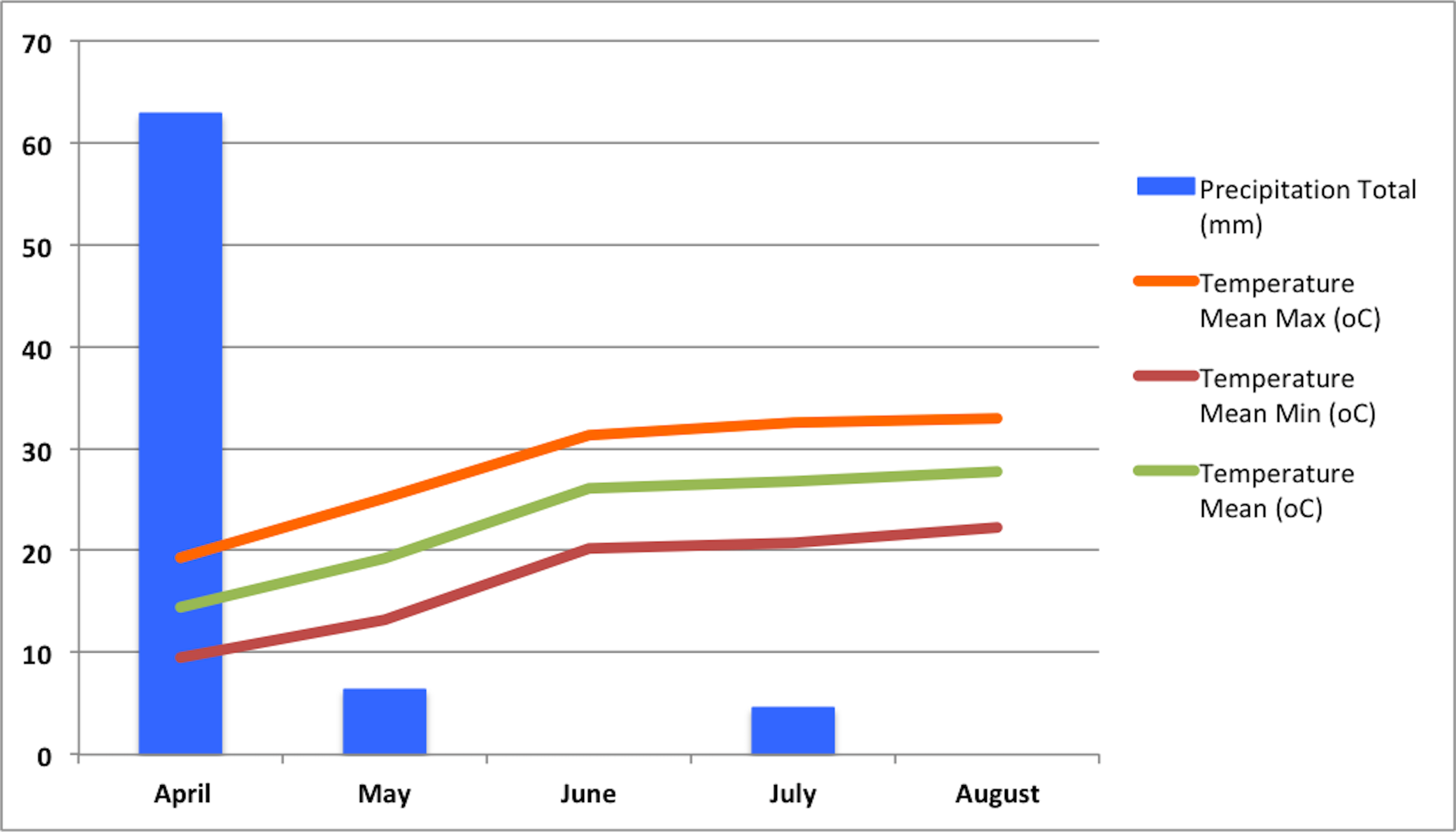


**Figure S1.** Monthly mean temperature and precipitation at Oropos during the experimental period.
